# Bioactive Peptides from Skipjack Tuna Cardiac Arterial Bulbs: Preparation, Identification, Antioxidant Activity, and Stability against Thermal, pH, and Simulated Gastrointestinal Digestion Treatments

**DOI:** 10.3390/md20100626

**Published:** 2022-09-30

**Authors:** Wei-Wei Cai, Xiao-Meng Hu, Yu-Mei Wang, Chang-Feng Chi, Bin Wang

**Affiliations:** 1 Zhejiang Provincial Engineering Technology Research Center of Marine Biomedical Products, School of Food and Pharmacy, Zhejiang Ocean University, Zhoushan 316022, China; 2 National and Provincial Joint Laboratory of Exploration, Utilization of Marine Aquatic Genetic Resources, National Engineering Research Center of Marine Facilities Aquaculture, School of Marine Science and Technology, Zhejiang Ocean University, Zhoushan 316022, China

**Keywords:** Skipjack tuna (*Katsuwonus pelamis*), cardiac arterial bulbs, peptide, antioxidant activity, stability

## Abstract

Cardiac arterial bulbs of Skipjack tuna (*Katsuwonus pelamis*) are rich in elastin, and its hydrolysates are high quality raw materials for daily cosmetics. In order to effectively utilizing Skipjack tuna processing byproducts-cardiac arterial bulbs and to prepare peptides with high antioxidant activity, pepsin was selected from six proteases for hydrolyzing proteins, and the best hydrolysis conditions of pepsin were optimized. Using ultrafiltration and chromatographic methods, eleven antioxidant peptides were purified from protein hydrolysate of tuna cardiac arterial bulbs. Four tripeptides (QGD, PKK, GPQ and GLN) were identified as well as seven pentapeptides (GEQSN, GEEGD, YEGGD, GEGER, GEGQR, GPGLM and GDRGD). Three out of them, namely the tripeptide PKK and the pentapeptides YEGGD and GPGLM exhibited the highest radical scavenging activities on 2,2-diphenyl-1-picrylhydrazyl (DPPH), hydroxyl, 2,2′-azino-bis-3-ethylbenzothiazoline-6-sulfonic acid (ABTS) and superoxide anion assays. They also showed to protect plasmid DNA and HepG2 cells against H_2_O_2_-induced oxidative stress. Furthermore, they exhibited high stability under temperature ranged from 20-100 °C, pH values ranged from 3-11, and they simulated gastrointestinal digestion for 240 min. These results suggest that the prepared eleven antioxidant peptides from cardiac arterial bulbs, especially the three peptides PKK, YEGGD, and GPGLM, could serve as promising candidates in health-promoting products due to their high antioxidant activity and their stability.

## 1. Introduction

Bioactive peptides (BPs) are usually composed of 2–20 amino acid (AA) residues and generated from plant, animal, and microorganism proteins by chemical degradation, biological fermentation, and enzymatic hydrolysis methods [1,2,3,4]. Beyond their well-recognized nutritional value, BPs have been turned out to be of great value in advancing human health because of their significant biological activities including anticancer [5,6], antioxidant [7], antihypertensive [8,9], antiinflammatory [10], hypolipidemic [11], immunoreglatory [6], anti-diabetic [12], and mineral binding activities [13]. Presently, global annualfish production is about 1.79 × 10^8^ tons and around 50% of these by-catches turn into processing byproducts in factories [14,15]. These fish byproducts lead to awful heavy handling problems, but inappropriate disposal will bring about environment contamination [1,16]. Therefore, many BPs were produced from those fish byproducts, including skins, heads, scales, and viscera [1,8,17,18].

Presently, oxidative stress damage and antioxidant ingredients have been widely studied in the field of food and medicine. Reactive oxygen species (ROS) generated under the unbalanced state of oxidative stress in cells can destroy some biological molecules, including lipids, proteins, enzymes, and DNA, which is proved to be associated with many physiological dysfunction and chronic diseases, such as hypertension, diabetes mellitus, hyperlipidemia, chronic nephropathy, neurodegenerative, cardiovascular, and inflammatory diseases [1,19,20,21,22]. Many synthetic antioxidants have served as drugs or additives to control oxidative damage. However, the dosage of those antioxidants has been rigidly controlled because of their potentiality for biological toxicity and DNA damage [23,24]. Recently, antioxidant peptides (APs) identified from different marine biological resources have shown great potential for treating a variety of diseases related to oxidative stress due to their significant antioxidant capacity of eliminating ROS, controlling lipid peroxidation, and improving the antioxidant system [1,25,26,27]. Skin gelatin peptides of Pacific cod could reduce and repair ultraviolet (UV)-radiated skin injuries by enhancing the activity of antioxidant enzymes and holding back the levels of nuclear factor kappa-light chain enhancer of B cells (NF-κB) and pro-inflammatory cytokines [28]. Ye et al. reported that BPs from monkfish muscle could mitigate high fat diet-induced hepatic steatosis in mice through up-regulating the content of phospho-AMP-activated protein kinase (p-AMPK), which further activate the nuclear factor erythroid-2-related factor 2 (Nrf2) pathway to increase the levels of heme oxygenase-1 (HO-1) and nicotinamide quinone oxidoreductase 1 (NQO1) [29]. Pentapeptide of LCGEC from tuna roe could activate the Nrf2 pathway to decrease the apoptosis of HaCaT cells induced by UV-B radiation. Furthermore, LCGEC could modulate the Nrf2 pathway after 6-week treatment in mice and inhibit the level of proinflammatory cytokines [30]. Oyster peptide fraction (molecular weight (MW) < 3500 Da) from trypsin hydrolysate of *Crassostrea talienwhanensis* showed significant hepatoprotective function on alcohol-caused hepatopathy in mice through activating the Nrf2 pathway to enhance the antioxidant capability in vivo and reducing the NF-κB level to inhibit the inflammatory response [31]. Therefore, food-derive APs showed outstanding application values in functional food, nutraceutical products, and pharmaceuticals in comparison with chemical antioxidants [7].

Skipjack tuna (*Katsuwonus pelamis*) with annual catches of 3.2 × 10^6^ tons is the most productive species of tuna. However, Skipjack tuna becomes the most important raw material for tuna canning industry due to its low value [32,33]. During tuna canning processing, about 50% Skipjack tuna were processed as byproducts, such as dark muscle, milt, bone/frame, scale, roe, head, and viscera [34,35,36]. Moreover, a variety of BPs were prepared from those byproducts and showed significant potential in the area of functional products. For example, the different peptides ICY, LSFR, IYSP, LPRS, SP, and VDRYF displayed significant blood pressure lowering activity by restraining angiotensin-I-converting enzyme (ACE) activity [33,36,37]. Other peptides, namely GPDGR, QDHKA, YEA, WMFDW, GADIVA, WMGPY, GAPGPQMV, EMGPA, DGPKGH, MLGPFPS, and AGPK presented the remarkable radical scavenging activity, lipid oxidation inhibition capability, and reducing power [24,38,39,40]. Peptides SMDV, GHHAAA, AEM, AEHNH, YVM, SVTEV, VRDQY, and PHPR displayed prominent cytoprotective ability on H_2_O_2_-damaged Chang liver cells [24,41]. Through the communication with enterprises, antioxidant peptides from tuna cardiac arterial bulbs may have a good market prospect in the development of daily cosmetics. However, we did not find the related research on the active peptides from this material in the literature. Therefore, to efficiently use tuna processing byproducts to produce functional substances, the objectives of the study were to prepare and identify APs from protein hydrolysate of skipjack tuna cardiac arterial bulbs. Moreover, the activity and stability of eleven isolated APs (TCP1 to TCP11) were analyzed and evaluated comprehensively.

## 2. Results

### 2.1. Preparation of Protein Hydrolysate of Skipjack Tuna Cardiac Arterial Bulbs

#### 2.1.1. Screening of Protease Species

**Figure 1:**
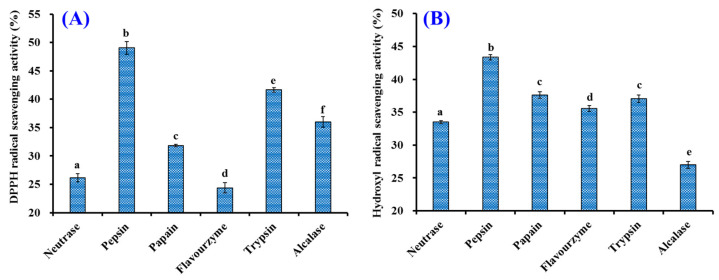
Influences of protease species on DPPH· (**A**) and HO· (**B**) scavenging activity of protein hydrolysates from Skipjack tuna cardiac arterial bulbs at 2.0 mg/mL. ^a–^^f^ Values with same letters indicate no significant difference (*p* > 0.05).

To more efficiently hydrolyze the proteins of tuna cardiac arterial bulbs, six proteases were selected to detect their hydrolytic ability (Figure 1). At 2.0 mg/mL, the scavenging activity of generated hydrolysate by pepsin on DPPH· and HO· was 49.06 ± 1.13% and 43.35 ± 0.42%, which was markedly higher than the activity of produced hydrolysates using Neutrase (26.18 ± 0.72% and 33.50 ± 0.24%), papain (31.84 ± 0.20% and 37.62 ± 0.50%), Flavourzyme (24.37 ± 0.90% and 35.56 ± 0.42%), trypsin (41.64 ± 0.42% and 37.05 ± 0.55%), and Alcalase (36.00 ± 0.93% and 26.99 ± 0.54%), respectively (*p* < 0.05). Therefore, pepsin was applied to produce the protein hydrolysate of tuna cardiac arterial bulbs.

#### 2.1.2. Pepsin Conditions Optimized by Single Factor Experiment

**Figure 2:**
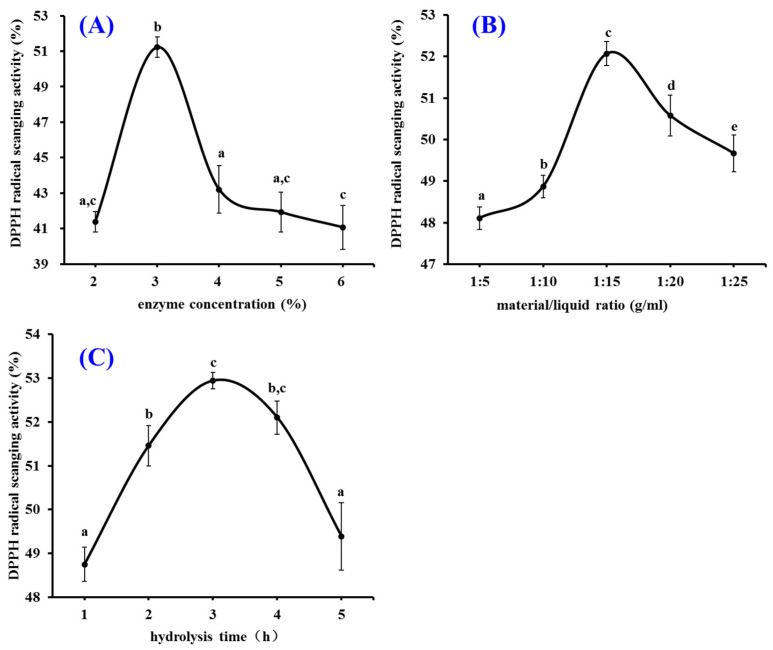
Effects of enzyme concentration (**A**), material/liquid ratio (**B**), and hydrolysis time (**C**) of pepsin on DPPH· scavenging activity of protein hydrolysates from tuna cardiac arterial bulbs at 2.0 mg/mL. ^a–^^e^ Values with same letters indicate no significant difference (*p* > 0.05).

The pepsin conditions including enzyme concentration, material/liquid ratio, and hydrolysis time on the influence of the radical scavenging rate of the hydrolysate of tuna cardiac arterial bulbs were optimized by the single-factor experiment (Figure 2). The data in Figure 2A revealed that enzyme concentration obviously affected the radical scavenging capability of prepared hydrolysates, and the DPPH· scavenging rate (49.23 ± 0.59%) of prepared hydrolysate at enzyme concentration of 3.0% was noticeably higher than those of prepared hydrolysates at other concentrations (*p* < 0.05). Figure 2B demonstrated that the radical scavenging rate of hydrolysates raised steadily when the material/liquid ratio ranged from 1:5 to 1:15 and reached the highest value (52.07 ± 0.29%) at the material/liquid ratio of 1:15. The results in Figure 2C suggested that the radical scavenging rates of prepared hydrolysates were significantly affected by the hydrolysis time, and the radical scavenging rate (52.94 ± 0.19%) of protein hydrolysate prepared for 3.0 h was significantly higher than those of prepared hydrolysates at other hydrolysis time (*p* < 0.05). Therefore, the range of hydrolysis conditions for pepsin was narrowed to 2–4%, 1:10–1:20, and 2–4 h for enzyme concentration, material/liquid ratio, and hydrolysis time, respectively.

#### 2.1.3. Pepsin Conditions Optimized by Response Surface Experiment 

According to the results in Figure 2, hydrolysis time (*X*_1_), material/liquid ratio (*X*_2_), and enzyme concentration (*X*_3_) were used to design the response surface experiment of 3-level, 3-factor factorial for further optimizing pepsin conditions, and the DPPH· scavenging rates of prepared hydrolysates of tuna cardiac arterial bulbs were shown in Table 1. Using design-Expert 8.0.6 Software (Stat-Ease Company, Minneapolis, America), the response values and the variables in Table 1 were fitted by regression, and the relationship between the DPPH· scavenging activity (*Y*) and the variables (hydrolysis time (*X*_1_), material/liquid ratio (*X*_2_), and enzyme concentration (*X*_3_)) was disclosed by the equation of quadratic multinomial regression, and the equation was as given below:

*Y*(%) = −16.55 + 16.67*X*_1_ + 2.04*X*_2_ + 17.53*X*_3_ + 0.05787*X*_1_*X*_2_ + 0.53241*X*_1_*X*_3_ − 0.26852*X*_2_*X*_3_ − 2.98032*X*_1_^2^ − 0.034954*X*_2_^2^ − 2.66782*X*_3_^2^ (1)

As shown in Table 2, *X*_1_, *X*_2_, *X*_3_, *X*_1_*X*_2_, *X*_1_*X*_3_, *X*_2_*X*_3_, *X*_1_^2^, *X*_2_^2^, and *X*_3_^2^ had significantly influences on DPPH· scavenging activity (*p* < 0.05). The results revealed that the influences of variances on the pepsin conditions had an interactive influence, not just a linear relation. The *R* Square (coefficient of determination, *R*^2^) of DPPH· scavenging activity was 0.8756, suggesting that 87.56% of the observed results would fit well to the regression equation. Additionally, the adjusted determination coefficient (*R*^2^_Adj_) was 0.7156, proving the high degree of consistency between the observed and predicted DPPH· scavenging activity; the low coefficient value (C.V.%) was 3.25, indicated that the mean value had little variation, high precision, and good reliability. Additionally, the difference of model (*p* = 0.0178 < 0.05) and Lack of Fit (*p* = 0.1001 > 0.05) showed that the model fit was excellent due to the small error of the regression equation. Therefore, the resulting model could be applied to correlation analysis and prediction of the relationship between the hydrolysate’s DPPH· scavenging activity and varying pepsin conditions. Moreover, the data in Table 2 indicated that the order of influential intensity of three variances on DPPH· scavenging activity was: hydrolysis time (*X*_1_) > enzyme concentration (*X*_3_) > material/liquid ratio (*X*_2_). Therefore, hydrolysis time has the most impact on DPPH· scavenging activity during the hydrolysis process.

**Figure 3:**
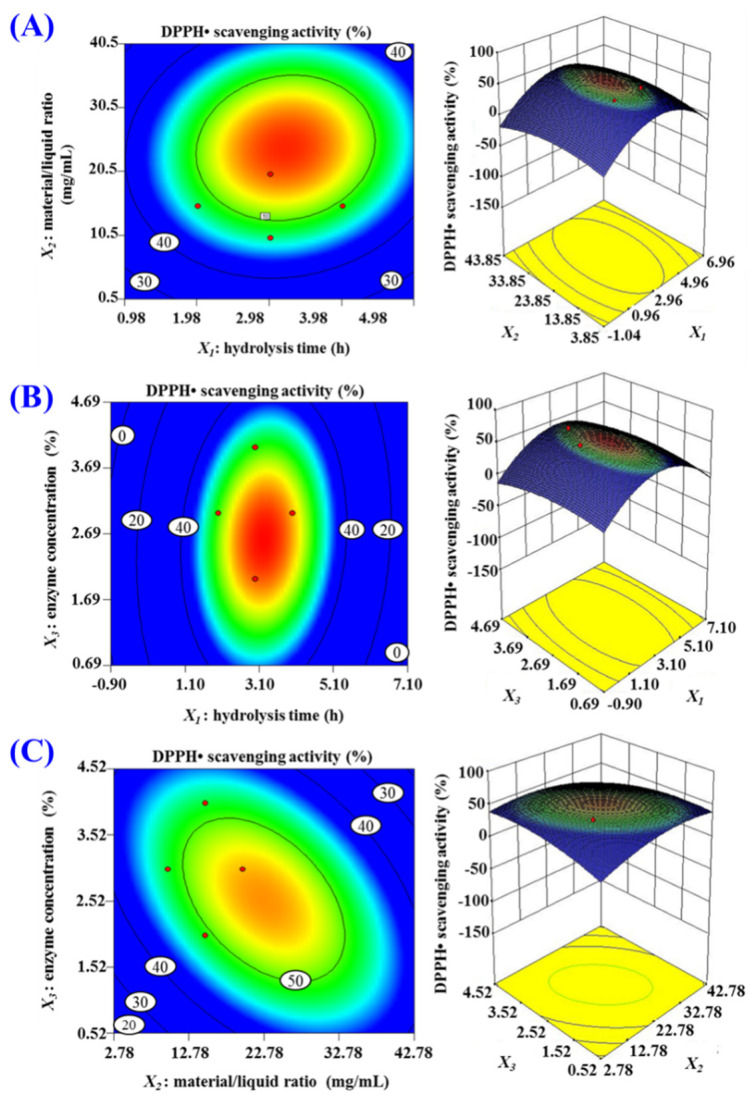
Response surface graph for DPPH· scavenging activity (%) as a function of (**A**) hydrolysis time (*X*_1_) and material/liquid ratio (*X*_2_), (**B**) hydrolysis time (*X*_1_) and enzyme concentration (*X*_3_), (**C**) material/liquid ratio (*X*_2_) and enzyme concentration (*X*_3_) during the hydrolysis of Skipjack tuna cardiac arterial bulbs with pepsin.

The response surface diagram was plotted on the basis of the multiple nonlinear regression equation (Figure 3). As shown in Figure 3A, DPPH· scavenging activity continuously increased when hydrolysis time (*X*_1_) was within a certain range, but DPPH· scavenging activity brought down instead of increasing when hydrolysis time (*X*_1_) was beyond this range. The impacting of material/liquid ratio (*X*_2_) and enzyme concentration (*X*_3_) presented the analogous tendency. In addition, the strength and significance of the interaction between two independent variables are reflected by the shape (elliptic or round) of contour map [36,37]. The contour maps of Figure 3B and 3C are elliptic, indicating the evident interaction between the two independent variables, but the contour maps of Figure 3A is nearer to round, indicating the indistinctive interaction between the two independent variables. These results indicated that the influence of *X*_1_*X*_3_ and *X*_2_*X*_3_ is more significant than that of *X*_1_*X*_3_. In accordance with the analysis by Design-Expert 8.0.6, the optimal conditions of pepsin for producing protein hydrolysate of tuna cardiac arterial bulbs were shown below: hydrolysis time 3.22 h, material/liquid ratio 1:20, and enzyme concentration 2.60%. Under the optimized conditions, the DPPH· scavenging rate of the hydrolysate (named as TCAH) of tuna cardiac arterial bulbs was 54.85%, which were in close proximity to the predicted 55.82%.

### 2.2. Preparation of APs from TCAH

#### 2.2.1. Ultrafiltration

**Figure 4:**
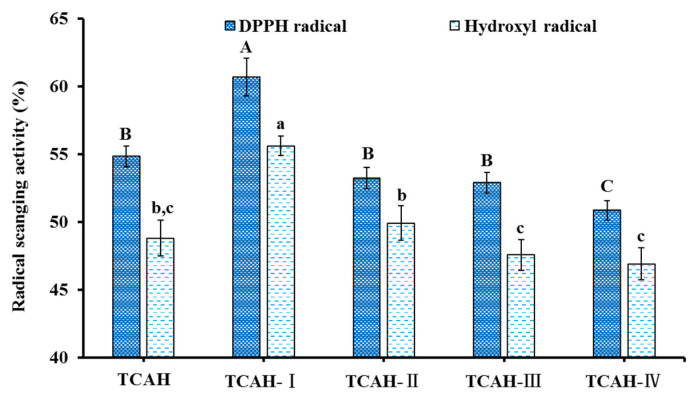
Radical scavenging activity of pepsin hydrolysate of Skipjack tuna cardiac arterial bulbs (TCAH) and its four fractions (TCAH-I to TCAH-IV). ^a–^^c^ Values and ^A–C^ values with same letters indicate no significant difference (*p* > 0.05).

The radical scavenging rates of TCAH and its four fractions TCAH-I (MW < 1.0 kDa), TCAH-II (1.0 < MW < 3.5 kDa), TCAH-III (3.5 < MW < 10 kDa), and TCAH-IV (MW > 10 kDa) at 2.0 mg/mL were determined (Figure 4). The data indicated that DPPH· and HO· scavenging rates of TCAH-I were 60.71 ± 1.39% and 55.62 ± 0.72%. The rates of TCAH-I were significantly higher than those of TCAH (54.85 ± 0.76% and 48.82 ± 1.32%), TCAH-II (53.26 ± 0.79% and 49.93 ± 1.27%), TCAH-III (52.91 ± 0.75% and 47.58 ± 1.13%), and TCAH-IV (50.87 ± 0.72% and 46.91 ± 1.18%) (*p* < 0.05). Hence, TCAH-I was selected for subsequent separation. 

#### 2.2.2. Chromatography 

According to the molecular size and polarity properties of BPs in the hydrolysate, multiple chromatographic methods were usually applied to prepare BPs from protein hydrolysates and their fractions [38,42]. 

**Figure 5:**
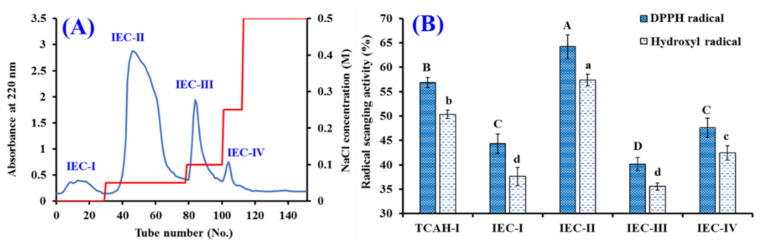
Chromatogram profile of TCAH-I isolated by DEAE-52 cellulose (**A**) and the radical scavenging activity of prepared subfractions (IEC-I to IEC-IV) from TCAH-I at 2 mg/mL (**B**). ^a–^^d^ Values and ^A^^–^^D^ values with same letters indicate no significant difference (*p* > 0.05).

Using a DEAE-52 cellulose column, four peptide fractions (IEC-I to IEC-IV) were isolated from TCAH-I (Figure 5A). At 2.0 mg/mL, DPPH· and HO· scavenging activities of IEC-II were 64.18 ± 2.45% and 58.33 ± 1.20% (Figure 5B). The activities of IEC-II were significantly higher than those of TCAH-I (60.71 ± 1.39% and 55.62 ± 0.72%), IEC-I (44.33 ± 2.00% and 37.62 ± 1.87%), IEC-III (40.15 ± 1.34% and 35.56 ± 0.74%), and IEC-IV (47.60 ± 2.00% and 42.46 ± 1.48%), respectively (*p* < 0.05).

**Figure 6:**
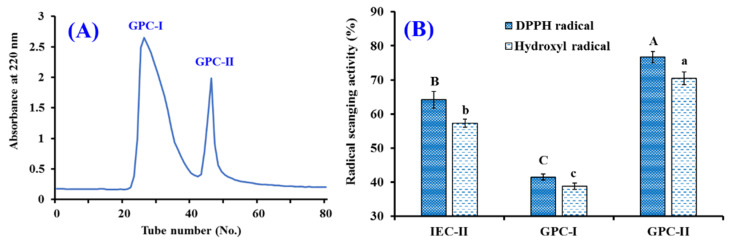
Chromatogram profile of IEC-II isolated by Sephadex G-25 (**A**) and the radical scavenging activity of prepared subfractions (GPC-I and GPC-II) from IEC-II at 2 mg/mL (**B**). ^a–^^c^ Values and ^A–C^ values with same letters indicate no significant difference (*p* > 0.05).

The subfraction of IEC-II was fractionated by Sephadex G-25 column, and two fractions (GPC-I and GPC-II) were purified in accordance with the chromatographic curves of IEC-II at 220 nm (Figure 6A). At 2.0 mg/mL, DPPH· and HO· scavenging rates of GPC-II were 76.71 ± 1.66% and 70.45 ± 1.84%, which were significantly higher than those of IEC-II (64.18 ± 2.45% and 57.33 ± 1.20%) and GPC-I (41.51 ± 0.83% and 38.84 ± 0.95%) (*p* < 0.05).

**Figure 7:**
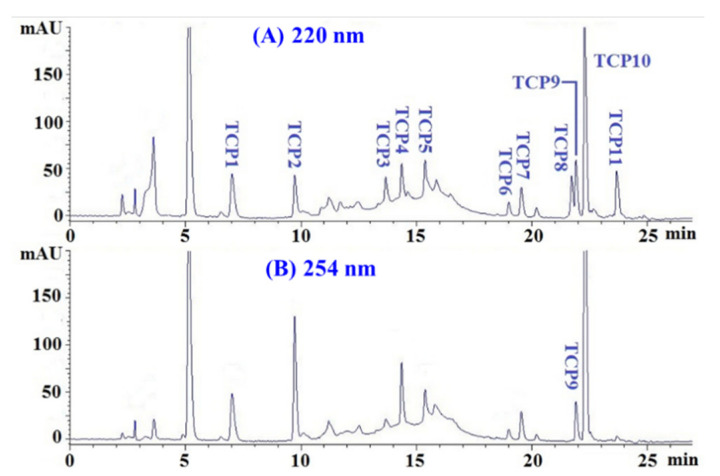
Elution profiles of GPC-II by RP-HPLC at 220 nm (**A**) and 254 nm (**B**).

Finally, GPC-II was isolated using RP-HPLC (Figure 7). On the basis of the elution profiles of GPC-II at 220 and 254 nm, 11 APs with retention time (min) of 7.05 (TCP1), 9.78 (TCP2), 13.68 (TCP3), 14.35 (TCP4), 15.38 (TCP5), 19.03 (TCP6), 19.57 (TCP7), 21.81 (TCP8), 21.95 (TCP9), 22.21 (TCP10), and 23.69 min (TCP11) were purified and lyophilized (Table 3). Then, 11 APs were enriched for structure identification.

### 2.3. Determination of the Amino Acid Sequences and MWs of TCP1 to TCP11 

**Figure 8:**
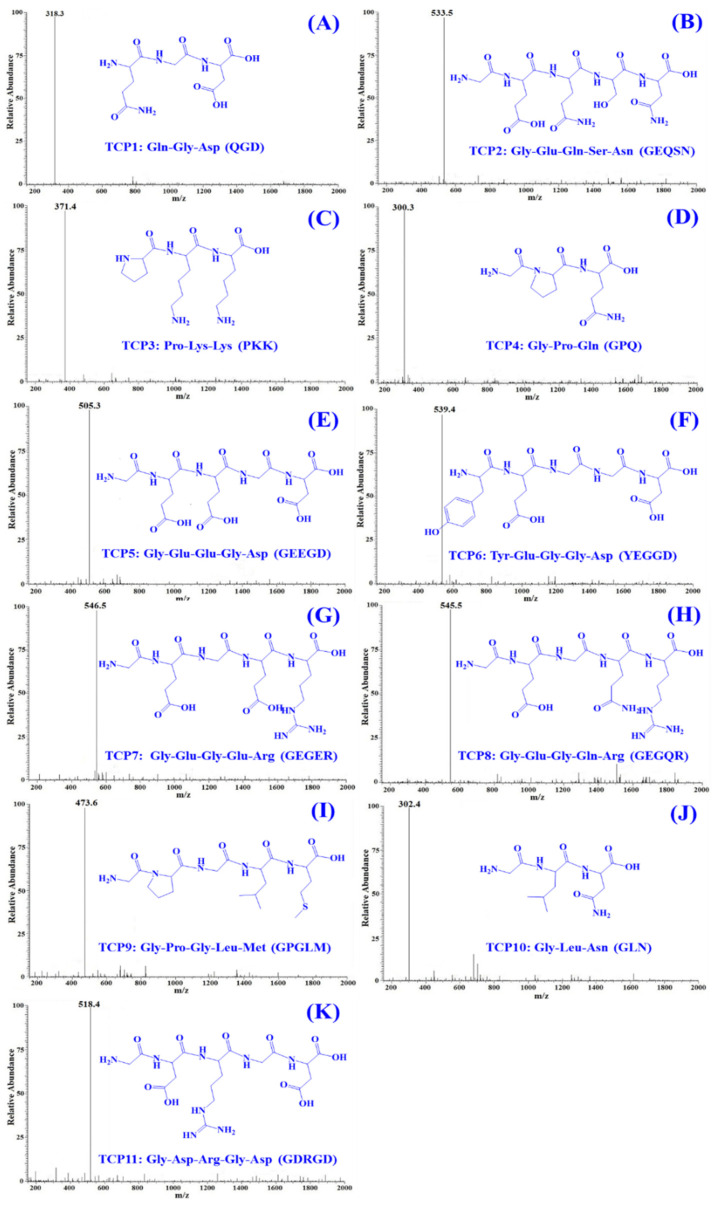
Mass spectrogram of 11 APs (TCP1 to TCP11) from hydrolysate of Skipjack tuna cardiac arterial bulbs (TCAH). (**A**) TCP1; (**B**) TCP2; (**C**) TCP3; (**D**) TCP4; (**E**) TCP5; (**F**) TCP6; (**G**) TCP7; (**H**) TCP8; (**I**) TCP9; (**J**) TCP10; (**K**) TCP11.

The amino acid sequences and MWs of TCP1 to TCP11 were measured using protein sequencer and ESI-MS. As shown in Table 3, the amino acid sequences of TCP1 to TCP11 were identified as Gln-Gly-Asp (QGD, TCP1), Gly-Glu-Gln-Ser-Asn (GEQSN, TCP2), Pro-Lys-Lys (PKK, TCP3), Gly-Pro-Gln (GPQ, TCP4), Gly-Glu-Glu-Gly-Asp (GEEGD, TCP5), Tyr-Glu-Gly-Gly-Asp (YEGGD, TCP6), Gly-Glu-Gly-Glu-Arg (GEGER, TCP7), Gly-Glu-Gly-Gln-Arg (GEGQR, TCP8), Gly-Pro-Gly-Leu-Met (GPGLM, TCP9), Gly-Leu-Asn (GLN, TCP10), and Gly-Asp-Arg-Gly-Asp (GDRGD, TCP11), respectively. The MWs of TCP1–TCP11 were determined as 318.28, 533.49, 371.48, 300.31, 505.43, 539.49, 546.53, 545.55, 473.59, 302.33, and 518.48 Da, respectively (Figure 8), which were quite consistent with their theoretical MWs (Table 3).

### 2.4. Antioxidant Activity of TCP1 to TCP11

#### 2.4.1. Radical Scavenging Activity of TCP1 to TCP11

As shown in Table 4, TCP3 with the EC_50_ value of 0.978 ± 0.006 mg/mL showed the highest DPPH· scavenging activity among 11 isolated peptides (TCP1 to TCP11), and other peptides with higher DPPH· scavenging activity were followed by TCP6 (1.062 ± 0.032 mg/mL), TCP9 (1.149 ± 0.039 mg/mL), and TCP4 (1.186 ± 0.008 mg/mL), respectively. Moreover, the EC_50_ values of TCP3, TCP4, TCP6, and TCP9 were also lower than those of APs from tuna roes (SGE: 1.342 mg/mL; VEP: 3.313 mg/mL) [24], pond loach meat (PSYV: 2.64 mg/mL) [43], *Tegillarca granosa* (TPP: 1.38 mg/mL) [44], Siberian sturgeon cartilages (GEYGFE, PSVSLT 1.27 mg/mL; IELFPGLP: 1.38 mg/mL) [42], *Mytilus edulis* (YPPAK: 2.62 mg/mL) [45], salmon pectoral fin (TTANIEDRR: 2.50 mg/mL) [46], and *Eucheuma cottonii* (YSKT: 1.71 mg/mL; FYKA: 2.56 mg/mL) [47]. However, the EC_50_ values of TCP3, TCP4, TCP6, and TCP9 were higher than those of APs from *E. cottonii* (YLL: 0.52 mg/mL) [47], blood cockle (WPPD: 0.36 mg/mL) [48], redlip croaker scales (GPEGPMGLE: 0.59 mg/mL, EGPFGPEG: 0.37 mg/mL, and GFIGPTE: 0.45 mg/mL) [49], and tuna milts (SVTEV: 0.89 mg/mL; VRDQY: 0.88 mg/mL) [50].

The data in Table 4 revealed that TCP3 (EC_50_: 1.158 ± 0.032 mg/mL) had the highest HO· scavenging activity among 11 APs (TCP1 to TCP11). The EC_50_ value of TCP3 was not significant lower than those of TCP6 (1.243 ± 0.027 mg/mL) and TCP9 (1.285 ± 0.016 mg/mL), but it was significantly lower than those of other eight isolated APs (*p* < 0.05). In addition, the EC_50_ values of TCP3, TCP6, and TCP9 on HO· were lower than those of APs from grass carp skin (VGGRP: 2.06 mg/mL) [51], Siberian sturgeon cartilages (IELFPGLP 1.63 mg/mL) [42], tuna roes (SGE: 2.76 mg/mL) [24], *T. granosa* (WLDPDG: 1.54 mg/mL) [48], and loach meat (PSYV: 2.64 mg/mL) [43]. However, the EC_50_ values of TCP3, TCP6, and TCP9 on HO· were higher than those of tuna milts (AEM: 0.456 mg/mL; QDHKA: 0.536 mg/mL; YEA: 0.476 mg/mL) [24], Pacific oyster (LKQELEDLLEKQE:0.046 mg/mL) [52], *Mytilus edulis* (YPPAK (0.228 mg/mL) [45], redlip croaker scales (GPEGPMGLE: 0.45 mg/mL) [26], giant squid (NADFGLNGLEGLA: 0.612 mg/mL) [53], *T. granosa* (WPPD: 0.38 mg/mL) [48], and Antarctic krill (SLPY: 0.826 mg/mL; EYEA 0.946 mg/mL) [54].

As shown in Table 4, TCP3 with the EC_50_ value of 0.188 ± 0.002 mg/mL showed the highest ABTS^+^· scavenging activity among 11 isolated APs, and other peptides with higher ABTS^+^· scavenging activity were followed by TCP6 (0.200 ± 0.002 mg/mL), TCP9 (0.216 ± 0.007 mg/mL), and TCP11 (0.226 ± 0.006 mg/mL), respectively. Moreover, the EC_50_ values of TCP3, TCP6, TCP9, and TCP11 were also lower than those of APs from tuna milts (GHHAAA: 1.12 mg/mL; SVTEV:1.02 mg/mL; VRDQY: 1.04 mg/mL) [41], *T. granosa* (QP: 1.38 mg/mL) [44], *Navodon septentrionalis* heads (GVPLT: 3.124 mg/mL) [20], blood cockle (WPPD: 0.54 mg/mL) [48], and *Scomberomorous niphonius* skins (YGPM: 0.82 mg/mL) [39]. However, the EC_50_ values of TCP3, TCP6, TCP9, and TCP11 were significant lower than that of positive control (0.097 ± 0.002 mg/mL) (*p* < 0.05).

Table 4 showed that EC_50_ values of TCP3, TCP6, TCP9, and TCP11 on O_2_^−^· was 0.924 ± 0.003, 0.933 ± 0.011, 0.969 ± 0.014, and 0.951 ± 0.008 mg/mL, which were significant lower than those of other eight isolated APs (*p* < 0.05). More than that, EC_50_ values of TCP3, TCP6, TCP9, and TCP11 were significant less than those of APs from tuna roes (SGE: 2.087 mg/mL; QEAE: 1.518 mg/mL; QAEP:2.916 mg/mL) [24], skate cartilages FIMGPY: 1.61 mg/mL) [55], blood cockle (EPLSD: 2.04 mg/mL) [48], *Miichthys miiuy* swim bladders (VPDDD: 4.11 mg/mL; GFYAA: 3.03 mg/mL) [15], and *Eucheuma cottonii* (YLL: 1.61 mg/mL; FYKA:1.91 mg/mL) [47]. However, EC_50_ values of TCP3, TCP6, TCP9, and TCP11 were significant higher than those of APs from *Eucheuma cottonii* (YAVT: 0.56 mg/mL) [47], *Mytilus edulis* (YPPAK: 0.072 mg/mL) [49], redlip croaker scales (GPEGPMGLE: 0.62 mg/mL, EGPFGPEG: 0.47 mg/mL; GFIGPTE: 0.74 mg/mL) [26], giant squid (NGLEGLK: 0.42 mg/mL) [53], *S. niphonius* skins (PYGAKG: 0.80 mg/mL; YGPM: 0.73 mg/mL) [39], Antarctic krill (EYEA: 0.793 mg/mL) [54], blood cockle (WPPD: 0.46 mg/mL) [48], and tuna roe (VDTR: 1.624 mg/mL; QEAE: 1.518 mg/mL) [24].

According to the results in Table 4, TCP3, TCP6, and TCP9 collectively showed minimal half clearance concentration (EC_50_), indicated that they have the strongest antioxidant activity among 11 isolated peptides. Given these results, TCP3, TCP6, and TCP9 were selected for the experiments of protective effects on Plasmid DNA and H_2_O_2_-damaged HepG2 cells.

#### 2.4.2. Protective Effects of TCP3, TCP6, and TCP9 on H_2_O_2_-damaged DNA 

**Figure 9:**
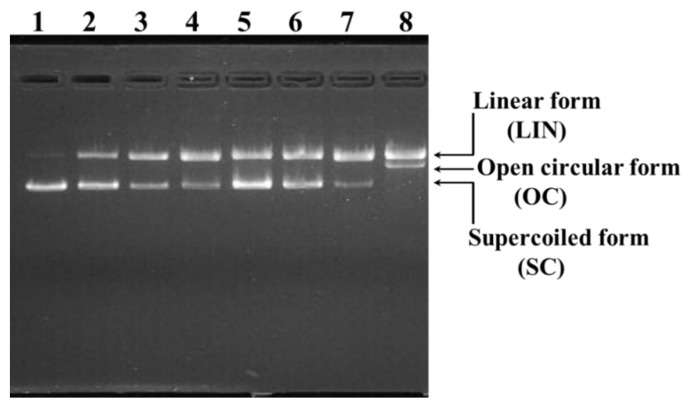
Protective effects of TCP3, TCP6, and TCP9 on H_2_O_2_-damaged plasmid DNA (pBR322DNA). Lane 1: the native pBR322DNA; Lane 2, DNA + FeSO_4_ + H_2_O_2_ + TCP9 (200 μM); Lane 3, DNA + FeSO_4_ + H_2_O_2_ + TCP6 (200 μM); Lane 4, DNA + FeSO_4_ + H_2_O_2_ + TCP3 (200 μM); Lane 5, DNA + FeSO_4_ + H_2_O_2_ + TCP9 (100 μM); Lane 6, DNA + FeSO_4_ + H_2_O_2_ + TCP6 (100 μM); Lane 7, DNA + FeSO_4_ + H_2_O_2_ + TCP3 (100 μM); Lane 8, pBR322DNA + FeSO_4_ + H_2_O_2_.

The protective effects of TCP3, TCP6, and TCP9 on H_2_O_2_-damaged pBR322DNA were evaluated and the results were showed in Figure 9. Under normal conditions, two phosphodiester chains of the plasmid DNA are primarily maintained the supercoiled (SC) form (Figure 9, lane 1). The open circular (OC) form was formed when one phosphodiester chain was cut off by HO· generated from the decomposition of H_2_O_2_, and the linear (LIN) form was produced if double-strand of DNA was break by the HO· (Figure 9, lane 8). Compared with the model group (Figure 9, lane 8), more SC form of the plasmid DNA was maintained in TCP3, TCP6, and TCP9 groups (Figure 9, lane 2–7), but the contents were significantly lower than those of normal control group (Figure 9, lane 1). The finding presented that TCP3, TCP6, and TCP9 has different degrees of protective functions on the plasmid DNA (pBR322DNA) damaged by the HO·, and the protective function of TCP9 was slightly stronger than those of TCP3 and TCP6. However, no significant concentration-effect relationship was observed between the protective effect and peptide concentration. The present results indicated that TCP3, TCP6, and TCP9 have potential capabilities of protecting pBR322DNA from oxidative damage. 

#### 2.4.3. Cytoprotective Effects of TCP3, TCP6, and TCP9 on H_2_O_2_-damaged HepG2 Cells

**Figure 10:**
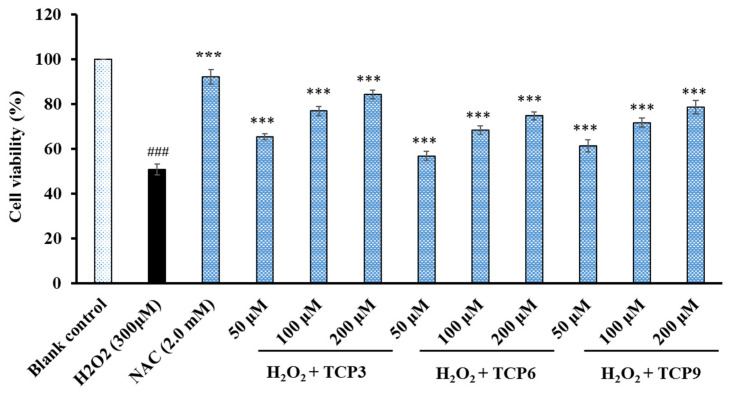
Effects of TCP3, TCP6, and TCP9 on the viability of H_2_O_2_-damaged HepG2 cells. N-Acetyl-L-Cysteine (NAC) was served as the positive control. ^###^ *p* < 0.001 vs. blank control group; *** *p* < 0.001 vs. model group.

Figure 10 indicated that TCP3, TCP6, and TCP9 concentration-dependently affected the viability of H_2_O_2_-damaged HepG2 cells when the peptide concentration increased from 50 to 200 μmol/L. At 200 μmol/L, the viability of TCP3, TCP6, and TCP9 groups was 81.26 ± 1.86%, 74.68 ± 1.69%, and 78.54 ± 3.06%, respectively. The data were significantly higher than that of model group (50.64 ± 2.43%), but significantly lower than that of N-Acetyl-L-Cysteine (NAC) group (90.06 ± 3.16%). Therefore, TCP3, TCP6, and TCP9 could provide a very good level of protective function to H_2_O_2_-damaged HepG2 cells by increasing the viability of HepG2 cells.

### 2.5. Stability of TCP1–TCP11

#### 2.5.1. Thermal Stability of TCP1–TCP11

**Figure 11:**
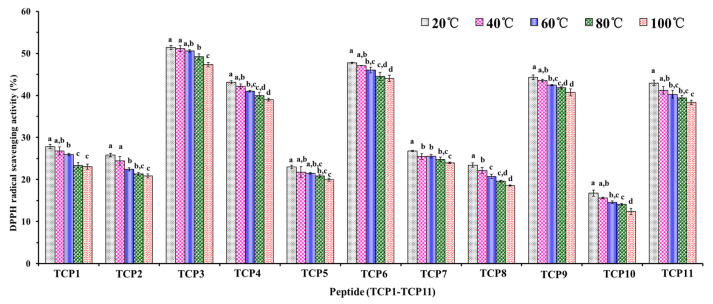
DPPH· scavenging activity of TCP1–TCP11 subjected to different thermal treatments for 60 min. ^a–d^ Values with same letters indicate no significant difference of same peptide (*p* > 0.05).

The DPPH· scavenging activity of TCP1–TCP11 under different temperature treatments (20, 40, 60, 80 and 100 °C) for 60 min was shown in Figure 11, the data indicated that thermal treatment, especially higher temperature (>60 °C) could affect the activity of TCP1–TCP11 (*p* < 0.05). In addition, the temperature tolerance of TCP1–TCP11 also showed some differences. Among 11 peptides, TCP7 and TCP8 presented the worst temperature tolerance because their radical scavenging activity already showed a significant difference between 20 and 40 °C. In addition, DPPH· scavenging activities of TCP3, TCP4, TCP6, TCP9, and TCP11 remained at a high level, and their activity only decreased by 7.95%, 9.62%, 7.73%, 8.08%, and 9.85% from 20 to 100 °C, respectively. Those data suggested that TCP3, TCP4, TCP6, TCP9, and TCP11 are suitable for use in products subjected to high temperature treatment due to their high activity and temperature tolerance.

#### 2.5.2. pH Stability of TCP1–TCP11 

**Figure 12:**
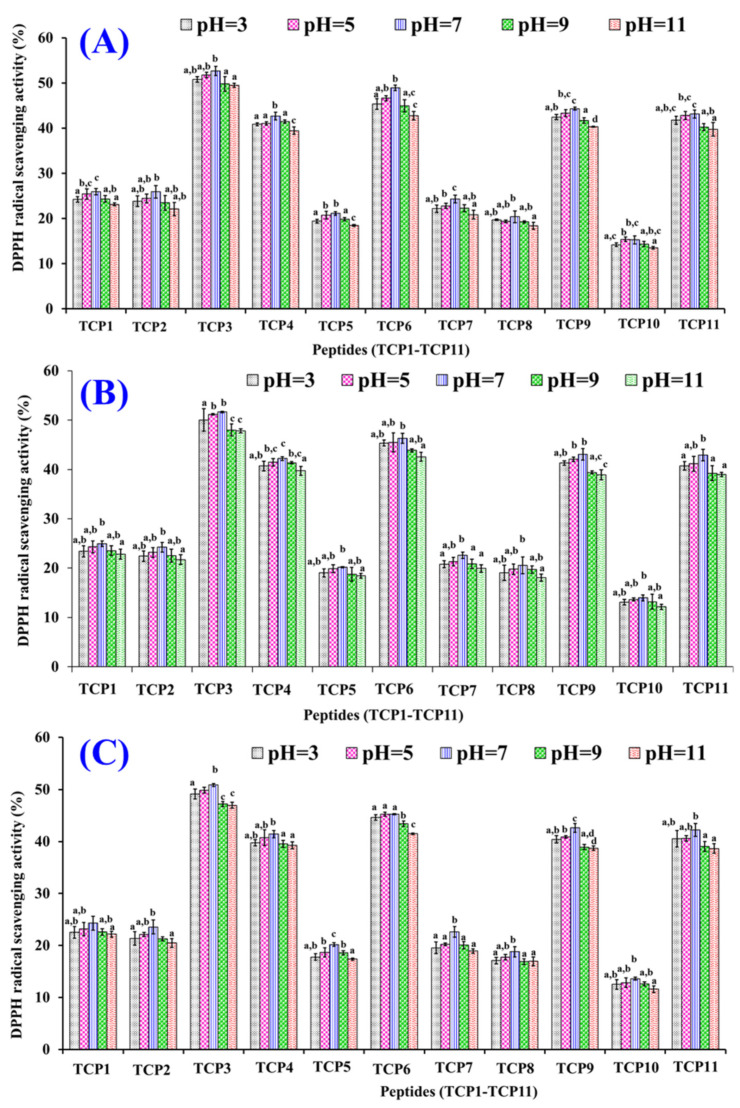
DPPH· scavenging activity of TCP1–TCP11 subjected to different pH treatments for 60 min (**A**), 120 min (**B**), and 180 min (**C**), respectively. ^a–c^ values with same letters indicate no significant difference of same peptide (*p* > 0.05).

Figure 12 indicated that the DPPH· scavenging rates of TCP1–TCP11 showed the same trend when they were treated with pH value ranged from 3 to 11. DPPH· scavenging rates of TCP1–TCP11 dealt with pH 7.0 treatment were significantly higher than those of TCP1–TCP11 subjected to pH 3.0 and 11.0 treatments. Additionally, the DPPH· scavenging rates of TCP1–TCP11 went down gradually with the time from 60 to 180 min. The data revealed that strong acid and alkali treatments had significantly adverse impacts on the radical scavenging rates of TCP1–TCP11. Moreover, DPPH· scavenging rates of TCP3, TCP4, TCP6, TCP9, and TCP11 remained at a high level at the designated pH values. At pH 7.0, their activity decreased by 3.36%, 3.02%, 7.51%, 3.88%, and 2.09% from 60 min to 180 min, respectively. Those data suggested that TCP3, TCP4, TCP6, TCP9, and TCP11 are suitable for use in products subjected to neutral solution environment.

#### 2.5.3. Stability of TCP1–TCP11 Subjected to Simulated Gastrointestinal (GI) Digestion 

**Figure 13:**
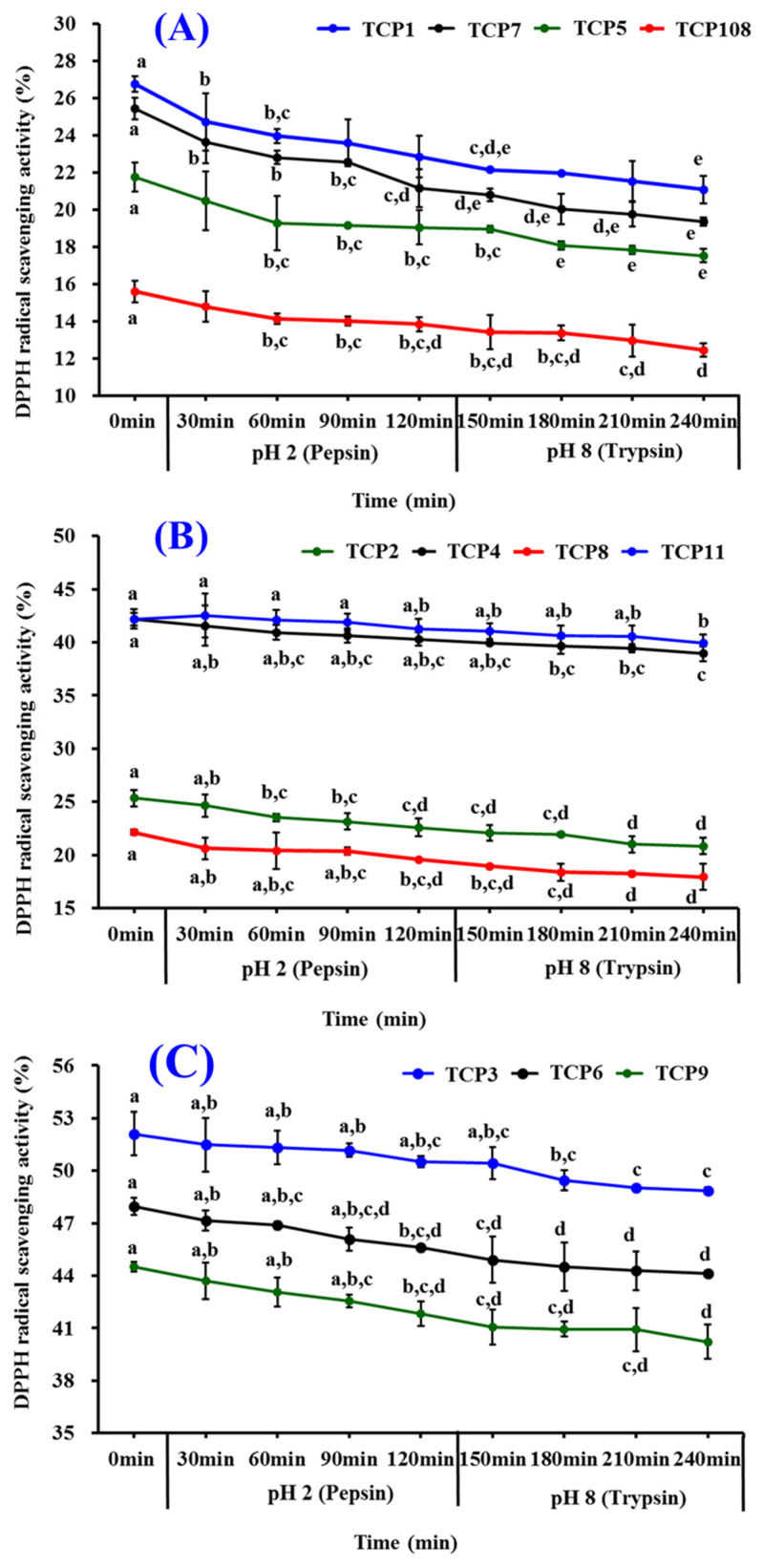
DPPH· scavenging activity of TCP1–TCP11 subjected to simulated GI digestion treatments from 0 to 240 min. (**A**): TCP1, TCP5, TCP7, and TCP10; (**B**): TCP2, TCP4, TCP8, and TCP11; (**C**): TCP3, TCP6, and TCP9. ^a–e^ Values with same letters indicate no significant difference of same peptide (*p* > 0.05).

Figure 13 presented the influences of simulated GI digestion on antioxidant activity of TCP1–TCP11, and the data revealed that DPPH· scavenging activities of TCP1–TCP11 were brought down gradually when the treating time of TCP1–TCP11 ranged from 0 to 240 min. Among 11 isolated peptides, the radical activity of TCP1 and TCP7 was most affected and decreased by 6.08% and 5.69% after simulated GI digestion for 240 min (Table 5). In addition, DPPH· scavenging activities of TCP3, TCP6, and TCP9 kept at a high level after simulated GI digestion for 240 min, and their activity decreased by 3.25%, 3.87%, and 4.30%, respectively. Those data suggested that TCP3, TCP6, and TCP9 have strong stability when they were treated with simulated GI digestion.

## 3. Discussion

### 3.1. Preparation of Antioxidant Peptides from Protein Hydrolysate of Tuna Cardiac Arterial Bulbs

Bioactive peptides are encrypted within the primary structure of proteins and remain inactive before being released by different hydrolysis methods [7]. Compared with the methods of solvent extraction, chemical degradation, and microbial fermentation, enzymatic hydrolysis is the most popular and effective method for preparing protein hydrolysates because of its simple operation characteristic and no damage to environment property [1,38]. The characteristics of proteases are the most crucial element affecting the composition and structure of BPs, which further influence the biological functions of protein hydrolysates [7,50,56]. Then, a variety of protease species, including Neutrase, papain, pepsin, Alcalase, Flavourzyme, chymotrypsin, and protamex, were screened, optimized, and used in the preparation of protein hydrolysates from multiple marine organisms and their by-products, such as Antarctic Krill [50], skate cartilages [5], miiuy croaker swim bladders [9], monkfifish muscle [21], Pacific cod skins [28], and tuna black muscles and scales [36,41]. The present results proved again that the specificity of proteases could significantly affect the peptide composition of protein hydrolysates, which are closely related to their antioxidant activity.

Protein hydrolysates are complex mixtures of peptides with different molecular size and amino acid composition, which are the most critical factors affecting the separating and purifying process of BPs [4,20]. Therefore, ultrafiltration technology usually acts as a powerful technique to enrich APs with small MWs from various hydrolysates [21,55]. In the experiment, the component (TCAH-I) with the lowest average molecular weight presented the strongest radical scavenging activity. The findings were consistent with the results in existing literature that small MW AP fractions of protein hydrolysates from different marine resources, such as bluefin leatherjacket skins [19], monkfish muscle [21], blue mussel [45], skate cartilages [55], Antarctic krill [50], and tuna milts [33] and heads [39], had the strongest radical scavenging abilities. In addition, chromatographic methods, including ion exchange chromatography, gel permeation chromatography, and RP-HPLC, are generally applied to purify peptides from hydrolysate fractions depending on their acidity/basicity, molecular size, and adsorption affinities [1,4]. On these methods, 11 antioxidant peptides (TCP1–TCP11) were purified from protein hydrolysate of tuna cardiac arterial bulbs and presented significant antioxidant activity.

### 3.2. Structure–Activity Relationship of TCP3, TCP6, and TCP9

Numerous APs have been purified from different protein resources, and several key elements, such as MW, amino acid sequence, and amino acid composition, are believed to play critical roles in their activities [57,58,59]. TCP3 is one tripeptide and TCP6 and TCP 9 are pentapeptides with MWs of 371.4, 539.4, and 473.6 Da, respectively. Smaller molecule facilitates the binding of TCP3, TCP6, and TCP9 to free radicals to play their scavenging functions [1,60]. Therefore, the low molecular weight peptides in TCP3, TCP6 and TCP9 easily enable free radicals to play a role and exert strong antioxidant activity.

Hydrophobic and aromatic amino acids were important for the activities of APs because they can promote the interaction between APs and radical species through increasing the solubility of the APs in lipids [38,61,62]. Leu residue contributed to the most important antioxidant functions of LKPGN, FMPLH, AEDKKLIQ and ILGATIDNSK from Antarctic Krill [50], miiuy croaker muscle [63], and chicken blood corpuscle [64]. Pro residue in VPR, IEPH, LDEPDPL, PHPR, PHH was good for their antioxidant ability because Pro residue could increase the flexibility of APs and directly eliminate singlet oxygen serving as proton/hydrogen donors [41,58,65]. Met residue could act as a reactive site in MWKVW, PMRGGGGYHY, SMDV, WMFDW, FMPLH, WMGPY, EMGPA, and ADMYW to get rid of radicals by transforming them into sulfoxide structures [41,63,66]. Aromatic groups of Tyr, Trp, and Phe residues in YPPAK, EDIVCW, MEPVW, YWDAW, and VRDQY can eliminate radicals by affording protons [21,41,45]. Therefore, Pro in TCP3, Tyr in TCP6, and Pro, Leu, and Met in TCP9 should play vital roles for their antioxidant functions.

Hydrophilic amino acid residues are also very crucial for the antioxidant activity of APs. Gly residue could keep high flexibility of the peptide skeleton and neutralize ROS by serving as the single hydrogen donor [40,63,67]. Then, Gly residues presented in TCP6 and TCP9 are helpful for their activity. In addition, Asp, Glu, Gln, and Lys residues made LHDVK, LDGP, EVGK, RCLQ, and TGVGTK from duck plasma have positive influences on their Fe^2+^ chelating capability [62]. Polar amino acid residues including the basic (Arg and Lys) and acidic (Glu and Asp) amino acid residues were highly regarded as the main conditions of antioxidant abilities of PELDW, LDEPDPLI, WPDHW, NTDGSTDYGILQINSR, MDLFTE, WPPD, FWKVV, TWKVV, and FGYDWW [48,63,67,68]. Furthermore, Wang et al. reported the similar results that Arg, Lys, Glu, and Asp were contributing to the activities of AEM, QDHKA, YEA, AEHNH, and YVM [24]. Therefore, Lys residues presented in TCP3 and Glu and Asp residues in TCP6 be very important to their radical scavenging activity.

### 3.3. Protective Functions of TCP3, TCP6, and TCP9 on H_2_O_2_-Damaged DNA and HepG2 Cells

In the organism, excessive ROS generated under oxidative stress can damage various cellular components [69]. This type of oxidative damage on DNA is mainly manifested in two ways: base modifications and DNA lacking a base, which are analogous to the result of DNA damage after ionizing radiation and usually accompanied by DNA fragmentation and cell apoptosis [70,71,72]. Therefore, DNA damage plays a causal role in the ROS-caused degradation processes, such as atherosclerosis, diabetes, cancer, premature aging, chronic inflammatory, and neurodegenerative diseases [73,74]. Therefore, protecting DNA from oxidative damage will have an important contribution to the prevention and treatment of chronic diseases [74,75]. The present finding (Figure 9) indicated that TCP3, TCP6, and TCP9 have potential capabilities of protecting DNA from oxidative damage.

In addition, superfluous ROS damage various bioactive macromolecules when cells are under the oxidative stress, which further lead to cell apoptosis [61]. Hu et al. reported that EDIVCW, MEPVW, and YWDAW could concentration-dependently defend HepG2 cells from H_2_O_2_-caused oxidative damage through activating intracellular antioxidant enzymes to decrease the levels of ROS [21]. GAERP, GEREANVM, and AEVG could protect HepG2 cells against H_2_O_2_ injury because they could decrease the malonaldehyde (MDA) content and increase the levels of intracellular antioxidant enzymes [76]. FWKVV, FMPLH, and FPYLRH from miiuy croaker could significantly bring down the oxidative injury of HUVECs caused by H_2_O_2_ because FWKVV, FMPLH, and FPYLRH could up-regulate the antioxidative enzyme activity and down-regulate the ROS level [22,23]. Decapeptide of APKGVQGPNG could decrease the ROS and NO contents in RAW 264.7 cells through up-regulating the Nrf-2 mediated HO-1 expression [61]. Therefore, the present study (Figure 10) suggested that TCP3, TCP6, and TCP9 might increase the viability of H_2_O_2-_damaged HepG2 cells.

According to the analysis of structure-activity relationship of TCP3, TCP6, and TCP9, their antioxidant activities are majorly dependent on the composition of amino acids and the length of the peptides. Small molecular size increases efficiency and leads to easier accessibility to the oxidant/antioxidant system. Hydrophobic amino acids increase their solubility at the water-lipid interface and thereby facilitate better interaction with free radicals (HO·, DPPH·, ABTS^+^·, and $O_{2}^{-}$·). Finally, antioxidant amino acids of TCP3, TCP6, and TCP9 change free radicals into a more stable structure or system to inhibit the propagation of the radical-mediated peroxidizing chain reaction through serving as a hydrogen donor, proton donor, and lipid peroxyl radical trap. In the assays of protective effects on H_2_O_2_-damaged Plasmid DNA and HepG2 cells, superfluous HO· was generated from the decomposition of H_2_O_2_. TCP3, TCP6, and TCP9 served similar functions to prevent or mitigate the damage caused by HO·. In addition, the cytoprotection of TCP3, TCP6, and TCP9 on H_2_O_2_-damaged HepG2 cells may be directly related to activating the Nrf2 pathway to enhance the activities of antioxidant enzymes. This question will be the focus of our lab’s subsequent research.

### 3.4. Stability of TCP1–TCP11

Thermal and acid/alkali stability are important processing parameters for peptides applied in functional products to lengthen the product shelf-life and expand their using fields. Tolerance property of APs on simulated GI digestion can help them to assess the metabolism rate and timeliness in vivo [48,77,78]. ATSHH treated at 50–90 °C partially lost its radical scavenging activity and suffered mild activity loss when ATSHH was dissolved in acidic (pH 2) and basic (pH 10–12) solutions [79]. MDLFTE and WPPD could not keep high antioxidant activity under high temperatures (>80 °C), basic pH value (pH > 9), or simulated GI digestion [48]. Similarly, the antioxidant activity of WMFDW, WMGPY, and EMGPA was significantly decreased when they were dealt with highly acid and alkali solutions and high temperature (>60 °C) [39]. However, WAFAPA, MYPGLA, VTAGLVGGGAGK, and PTGNPLSP have strong stability when they are treated with heat (25–100 °C) and pH values (3–11) [80,81]. EYEA, QYPPMQY, and SLPY isolated from Antarctic Krill showed a high degree of stability when the temperature was lower than 80 °C, pH values ranged from 6-8, and the time of simulated GI digestion was for 180 min [54]. In the experiment, DPPH· scavenging rates of TCP3, TCP6, and TCP9 descended by no more than 8.08%, 7.51%, and 4.30%, respectively, under high temperature (100 °C), pH values ranged from 3–11, and simulated GI digestion for 240 min, which reflected that TCP3, TCP6, and TCP9 should be stable under high thermal food processing and can maintain high antioxidant activity in acid and basic solutions. Furthermore, TCP3, TCP6, and TCP9 could keep their bioactivity and bioavailability in vivo because they are not susceptible to being degraded by GI digestive enzymes. 

## 4. Materials and Methods

### 4.1. Materials and Chemical Reagents

Cardiac arterial bulbs of Skipjack tuna were friendship provided by Jiri Food Co., Ltd. (Ningbo, China). HepG2 cells were bought from the Chinese Academy of Sciences (Shanghai, China). Trypsin, trifluoroacetic acid (TFA), N-Acetyl-l-Cysteine (NAC), DPPH, ABTS, methylthiazolyldiphenyl-tetrazolium bromide (MTT), pepsin, and papain were purchased from Sigma-Aldrich (Shanghai, China) Trading Co., Ltd. (Shanghai, China). Neutrase, Alcalase, and Flavourzyme were purchased from Shanghai Jingchun Biochemical Technology Co., Ltd. (Shanghai, China). DEAE-52 cellulose and Sephadex G-25 were purchased from Shanghai Yuanju Bio-Tech Co., Ltd. (Shanghai, China). Peptides of TCP1 to TCP11 (>98%) were synthesized in China Peptides Co., Ltd. (Suzhou, China).

### 4.2. Preparation of Protein Hydrolysate of Cardiac Arterial Bulbs (TCAH)

#### 4.2.1. Screening of Protease Species

The enzymatic hydrolysis process of tuna cardiac arterial bulbs was carried out according to the previous literature with a slight modification [24]. Firstly, the cardiac arterial bulbs were defrosted, crushed to pieces, and degreased using isopropanol. In brief, isopropanol was added into the pieces of cardiac arterial bulbs with a liquid/solid ratio of 5:1 (*v/w*) and continuously stirred for 60 min at 25 °C. Afterwards, the cardiac arterial bulbs solution was centrifuged at 6000× *g* for 20 min and the resulted cardiac arterial bulbs were defatted twice according to the above procedure. Finally, the resulted cardiac arterial bulbs were dried at 35 °C.

The defatted solutions of Skipjack tuna cardiac arterial bulbs were hydrolyzed separately using different proteases under the known conditions, including papain (pH 7.0, 55 °C), pepsin (pH 2.0, 37 °C), Flavourzyme (pH 7.0, 50 °C), trypsin (pH 7.8, 37 °C), Alcalase (pH 9.0, 50 °C), and Neutrase (pH 7.0, 50 °C) with enzyme concentration of 2.0%. Thereafter, the hydrolysis solutions were put in boiling water bath for 15 min and centrifuged at 12,000× *g* at 4 °C for 15 min. The resulting supernatant was lyophilized and stored at -20 °C. The protein hydrolysate of tuna cardiac arterial bulbs produced by pepsin showed the highest radical scavenging activity.

#### 4.2.2. Optimization of Hydrolysis Conditions of Pepsin

The hydrolysis conditions of pepsin were designed to optimize by a single factor experiment. Enzyme concentration (2%, 3%, 4%, 5%, and 6%), material/liquid ratio (1:5, 1:10, 1:15, 1:20, and 1:25), and hydrolysis time (1, 2, 3, 4, and 5 h) were selected as the process conditions for detection.

According to the above results, response surface methodology was applied to evaluate the effects of independent variables (*X*_1_, hydrolysis time; *X*_2_, material/liquid ratio; *X*_3_, enzyme concentration) on DPPH· scavenging activity [36,37]. Three levels (*X*_1_: 2, 3, and 4 h; *X*_2_: 1:10, 1:5, and 1:20; *X*_3_: 2.0, 3.5 and 4.0%) were chosen to evaluate the affecting of three variables on DPPH· scavenging rate. The hydrolysate of tuna cardiac arterial bulbs generated under the optimized enzymolysis conditions was referred to as TCAH. 

### 4.3. Preparation of APs from TCAH

**Figure 14:**
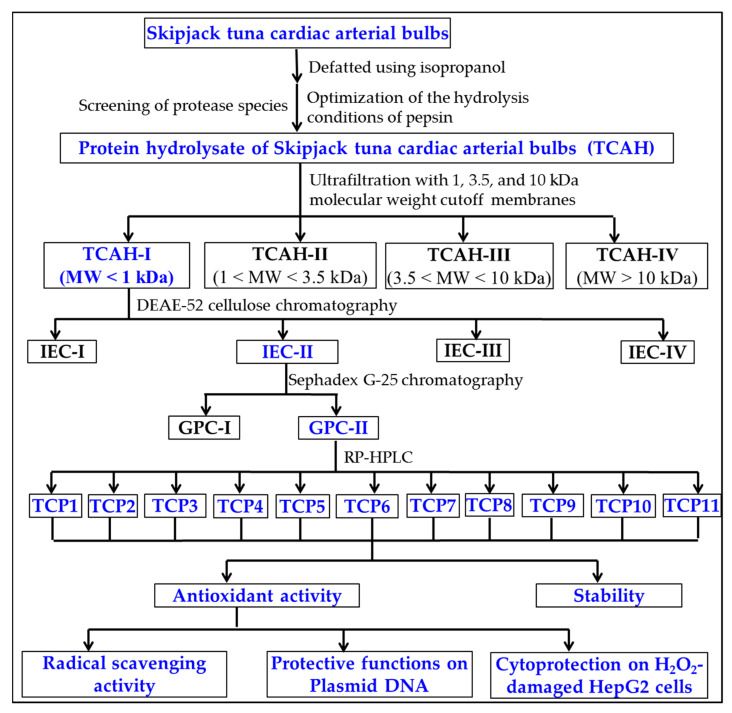
Flow diagram of isolating APs from protein hydrolysate (TCAH) of tuna cardiac arterial bulbs prepared by pepsin.

APs were purified from TCAH using the following designed process (Figure 14).

#### 4.3.1. Ultrafiltration of TCAH 

TCAH was fractionated using cut-off membranes with MWs of 1, 3.5 and 10 kDa, and four peptide fractions of TCAH-I (MW < 1.0 kDa), TCAH-II (1.0 < MW < 3.5 kDa), TCAH-III (3.5 < MW < 10 kDa), and TCAH-IV (MW > 10 kDa) were prepared. TCAH-I showed the highest radical scavenging activity.

#### 4.3.2. Purification of APs from TCAH-I by Chromatography Methods

TCAH-I solution (6 mL, 50.0 mg/mL) was injected into the DEAE-52 cellulose column (3.8 × 150 cm) pre-treated by deionized water, and the column was eluted separately using 1000 mL deionized water and three kinds of NaCl solutions (0.05, 0.10 and 0.25 mol/L) in succession at a flow rate of 3.0 mL/min. Then, four fractions (IEC-I to IEC-IV) were collected according to the chromatogram at 220 nm and freeze-dried.

IEC-II solutions (5 mL, 50.0 mg/mL) were loaded into the Sephadex G-25 column (2.6 cm × 120 cm) and eluted using ultrapure water. The eluent from the column at a flow rate of 0.8 mL/min was collected each 2 min, and two fractions (GPC-I and GPC-II) were collected on the basis of the chromatographic curves at 220 nm.

GPC-II was purified using an HPLC column of Zorbax, SB C-18 (4.6 × 250 mm, 5 µm). The HPLC column was eluted by a linear gradient of acetonitrile (0–50% in 0–30 min) in 0.1% TFA. The eluent from HPLC column with a flow rate of 1.0 mL/min was detected at 220 and 254 nm. Finally, 11 APs (TCP1 to TCP11) were prepared on the HPLC chromatograms at 220 and 254 nm and freeze-dried.

### 4.4. Identification of TCP1 to TCP11 

The amino acid sequences of TCP1 to TCP11 isolated from tuna cardiac arterial bulbs were determined by a 494 protein sequencer of Applied Biosystems (Perkin Elmer Co., Ltd. Foster City, CA, USA). The MWs of TCP1 to TCP11 were determined by a Q-TOF mass spectrometer with an ESI source (Micromass, Waters, Milford, MA, USA).

### 4.5. Antioxidant Activity of TCP1 to TCP11 from Tuna Cardiac Arterial Bulbs

#### 4.5.1. Radical Scavenging Activity 

The scavenging activity of TCP1 to TCP11 from tuna cardiac arterial bulbs on DPPH·, HO·, ABTS^+^·, and $O_{2}^{-}$· was measured according to the previous methods [45,49]. Half clearance concentration (EC_50_ value) was defined as the sample concentration resulted in a 50% decline of the initial radical concentration [82].

#### 4.5.2. Protective Effects of TCP3, TCP6, and TCP9 on Plasmid DNA

The protective effects of TCP3, TCP6, and TCP9 from tuna cardiac arterial bulbs on supercoiled plasmid DNA (pBR322) were measured according to the previous method [67]. Briefly, 5 µL of PBS (10 mM, pH 7.4), 2 µL of FeSO_4_ (1.0 mM), 1µL of pBR322 (0.5 µg), 5 µL of the AP (TCP3, TCP6, or TCP9, respectively), and 2 µL of H_2_O_2_ (1.0 mM) were successively mixed to prepare the reaction solution. A volume of 15 µL of prepared reaction mixtures were incubated at 37 °C for 30 min. Afterwards, 2 µL of loading buffer (containing glycerol (50%, *v/v*), EDTA (40 mM), and bromophenol blue (0.05%)) were added into the reaction mixtures to terminate the reaction of H_2_O_2_ and FeSO_4_. Then, the mixtures were separated subsequently by 1% agarose gel electrophoresis (containing 0.5 µg/mL EtBr) at 60 V for 50 min. Finally, the DNA in the agarose gel was photographed and recorded under UV light.

#### 4.5.3. Cytoprotection of TCP3, TCP6, and TCP9 on H_2_O_2_-damaged HepG2 Cells 

The assay was carried out according to the method described by Hu et al. [21], and the H_2_O_2_ concentration of 300 µM was applied to establish the oxidative damage model of HepG2 cells. Briefly, the HepG2 cells with density of 1 × 10^5^ cells/well were incubated in a 96-well plate for 24 h. The supernatant in the 96-well plate was aspirated and 100 µL of peptide samples (50.0, 100.0, or 200.0 µM, respectively) were added into the protection groups respectively for incubating 8 h. After removing peptides, H_2_O_2_ with the final concentration of 300 µM was joined in the protection and model groups. After incubating for 24 h, the wells were rinsed twice with PBS and the MTT with the final concentration of 0.5 mg/mL was added into. After incubating for 4 h, the formazan crystals formed by active cells were soluble in 150 µL of DMSO and OD_570 nm_ of the solution was determined. Finally, the cell viability was calculated using the equation:

Cell viability = (OD_sample_/OD_control_) × 100% (2)

### 4.6. Stability of TCP1 to TCP11 from Tuna Cardiac Arterial Bulbs

The stability of TCP1 to TCP11 from tuna cardiac arterial bulbs was determined according to the previous method with a slight modification [67]. The DPPH· scavenging activity (%) of TCP1 to TCP11 at 2.0 mg/mL were measured at the set conditions to evaluate their stability.

The thermostability of TCP1 to TCP11 at 20, 40, 60, 80, or 100 °C for 60 min was analyzed in water bath. The influences of pH values of 3, 5, 7, 9, or 11 were employed to estimate the acid and alkali stability properties of TCP1 to TCP11 at 25 °C, and the detecting time was set to 60, 120, and 180 min, respectively. The influence of simulate GI digestion on the stability of TCP1 to TCP11 was evaluated by the two-stage digestion model. In short, APs (TCP1 to TCP11) were separately treated with pepsin for 120 min and trypsin for 120 min.

### 4.7. Statistical Analysis

All the data are expressed as the mean ± SD (*n* = 3). The experimental data were analyzed by an ANOVA test using SPSS 19.0. (IBM, Palo Alto, America) Significant differences between the means of parameters were determined by Duncan’s multiple range test (*p* < 0.05).

## 5. Conclusions

In summary, the conditions of pepsin for hydrolyzing the protein of Skipjack tuna (*Katsuwonus pelamis*) processing byproducts-cardiac arterial bulbs were optimized, and 11 peptides (TCP1 to TCP11) were purified from the protein hydrolysate of tuna cardiac arterial bulbs and identified as QGD, GEQSN, PKK, GPQ, GEEGD, YEGGD, GEGER, GEGQR, GPGLM, GLN, and GDRGD, respectively. PKK, YEGGD, and GPGLM exhibited high radical scavenging activity and significant protective function on Plasmid DNA and HepG2 cells against H_2_O_2_-induced oxidative stress. Furthermore, PKK, YEGGD, and GPGLM had high stability under heat, acid/alkali, and simulated GI digestion treatments. Therefore, the obtained results provide a good chance for tuna processing byproducts-cardiac arterial bulbs as the materials to generate BPs, and the prepared APs could serve as promising antioxidant ingredients applied in health-promoting products. Moreover, further *in vivo* experiments will be performed in our laboratory for studying the mechanisms of APs, that use signal transduction pathway of Nrf2.

## Figures and Tables

**Table 1:** Box-Behnken design and experimental results of response surface methodology.

Run	Independent Variables		Dependent Variables	
	*X*_1_ (Hydrolysis Time/h)	*X_2_* (Material/Liquid Ratio/mg/mL)	*X*_3_ (Enzyme Concentration/%)	*Y* (DPPH· Scavenging Activity, %)
1	2	1:10	3	48.11
2	4	1:10	3	48.57
3	2	1:20	3	49.73
4	4	1:20	3	51.35
5	2	1:15	2	47.97
6	4	1:15	2	50.84
7	2	1:15	4	43.39
8	4	1:15	4	48.39
9	3	1:10	2	45.94
10	3	1:20	2	53.67
11	3	1:10	4	48.53
12	3	1:20	4	50.89
13	3	1:15	3	52.42
14	3	1:15	3	53.53
15	3	1:15	3	52.28
16	3	1:15	3	53.34
17	3	1:15	3	54.92

**Table 2:** ANOVA for response surface quadratic model: estimated regression model of relationship between DPPH· scavenging activity and pepsin conditions.

Source	Sum of Squares	df	Mean Square	*F*-Value	*p*-Value	
Model	130.89	9	14.54	5.47	0.0178	significant
*X*_1_-hydrolysis time	26.87	1	26.87	10.11	0.0155	
*X*_2_-material-to-liquid ratio	0.41	1	0.41	0.15	0.7068	
*X*_3_-enzyme concentration	1.23	1	1.23	0.46	0.5180	
*X*_1_ *X*_2_	0.33	1	0.33	0.13	0.7330	
*X*_1_ *X*_3_	1.13	1	1.13	0.43	0.5345	
*X*_2_ *X*_3_	7.21	1	7.21	2.71	0.1435	
*X*_1_^2^	37.40	1	37.40	14.07	0.0072	
*X*_2_^2^	3.22	1	3.22	1.21	0.3077	
*X*_3_^2^	29.97	1	29.97	11.28	0.0121	
Residual	18.60	7	2.66			
Lack of Fit	14.11	3	4.70	4.19	0.1001	not significant
Pure Error	4.49	4	1.12			
Cor Total	149.49	16			0.0178	
*R*^2^ = 0.8756	*R*^2^_Adj_ = 0.7156	CV(%) = 3.25	Adeq Precision = 7.073			

**Table 3:** Retention time, amino acid sequences, and molecular weights (MWs) of 11 APs (TCP1–TCP11) from protein hydrolysate (TCAH) of Skipjack tuna cardiac arterial bulbs.

	Retention Time (min)	Amino Acid Sequence	Observed MW/Theoretical MW (Da)
TCP1	7.05	Gln-Gly-Asp (QGD)	318.3/318.3
TCP2	9.78	Gly-Glu-Gln-Ser-Asn (GEQSN)	533.5/553.5
TCP3	13.68	Pro-Lys-Lys (PKK)	371.4/371.5
TCP4	14.35	Gly-Pro-Gln (GPQ)	300.3/300.3
TCP5	15.38	Gly-Glu-Glu-Gly-Asp (GEEGD)	505.3/505.4
TCP6	19.03	Tyr-Glu-Gly-Gly-Asp (YEGGD)	539.4/539.5
TCP7	19.57	Gly-Glu-Gly-Glu-Arg (GEGER)	546.5/546.5
TCP8	21.81	Gly-Glu-Gly-Gln-Arg (GEGQR)	545.5/545.6
TCP9	21.95	Gly-Pro-Gly-Leu-Met (GPGLM)	473.6/473.6
TCP10	22.21	Gly-Leu-Asn (GLN)	302.4/302.3
TCP11	23.69	Gly-Asp-Arg-Gly-Asp (GDRGD)	518.4/518.5

**Table 4:** EC_50_ values of 11 antioxidant peptides (TCP1 to TCP11) from protein hydrolysate of Skipjack tuna cardiac arterial bulbs (TCAH) on HO·, DPPH·, ABTS^+^·, and O_2_^−^·, respectively.

EC_50_ (mg/mL)				
	DPPH·	HO·	ABTS^+^·	O_2_^−^·
TCP1	1.867 ± 0.018 ^a^	1.931 ± 0.006 ^a^	0.273 ± 0.013 ^a^	1.558 ± 0.032 ^a^
TCP2	2.054 ± 0.021 ^b^	2.123 ± 0.031 ^b^	0.529 ± 0.003 ^b^	1.857 ± 0.002 ^b^
TCP3	0.978 ± 0.006 ^c^	1.158 ± 0.032 ^c^	0.188 ± 0.002 ^c^	0.924 ± 0.003 ^c^
TCP4	1.186 ± 0.008 ^d, g^	1.307 ± 0.006 ^d^	0.269 ± 0.013 ^a^	1.063 ± 0.007 ^d^
TCP5	2.296 ± 0.011 ^e^	2.744 ± 0.108 ^e^	1.447 ± 0.016 ^d^	2.980 ± 0.012 ^e^
TCP6	1.062 ± 0.032 ^c, d^	1.243 ± 0.027 ^c, d^	0.200 ± 0.002 ^c, e^	0.933 ± 0.011 ^c^
TCP7	1.964 ± 0.031 ^a, b^	2.049 ± 0.018 ^b^	0.407 ± 0.008 ^f^	1.640 ± 0.055 ^f^
TCP8	2.257 ± 0.038 ^e^	2.505 ± 0.035 ^f^	1.218 ± 0.005 ^g^	2.143 ± 0.040 ^g^
TCP9	1.149 ± 0.039 ^d, g^	1.285 ± 0.016 ^c,d^	0.216 ± 0.007 ^e, h^	0.969 ± 0.014 ^c^
TCP10	3.204 ± 0.177 ^f^	3.556 ± 0.042 ^g^	1.641 ± 0.003 ^i^	3.022 ± 0.015 ^e^
TCP11	1.214 ± 0.036 ^g^	1.739 ± 0.050 ^h^	0.226 ± 0.006 ^h^	0.951 ± 0.008 ^c^
GSH	0.085 ± 0.002 ^h^	0.504 ± 0.011 ^i^	0.097 ± 0.002 ^j^	0.278 ± 0.016 ^h^

^a–j^ Values with same letters indicate no significant difference (*p* > 0.05).

**Table 5:** Declined percentage of DPPH· scavenging activity of TCP1–TCP11 subjected to simulated GI digestion treatments from 0 to 240 min.

Peptides	Declined Percentage	Peptides	Declined Percentage
TCP1	5.69%	TCP7	6.08%
TCP2	4.49%	TCP8	4.21%
TCP3	3.25%	TCP9	4.30%
TCP4	3.22%	TCP10	3.15%
TCP5	4.25%	TCP11	2.65%
TCP6	3.87%		

## Data Availability

Data are contained within the article.

## Abbreviations

BP, bioactive peptide; AP, antioxidant peptide; ROS, reactive oxygen species; UV, ultraviolet; NF-κB, nuclear factor kappa-light chain enhancer of B cells; HUVEC, human umbilical vein endothelial cell; p-AMPK, phospho-AMP-activated protein kinase; Nrf2, nuclear factor erythroid-2-related factor 2; HO-1, heme oxygenase-1; NQO1, nicotinamide quinone oxidoreductase 1; ACE, angiotensin-I-converting enzyme; TFA, trifluoroacetic acid; GI: gastrointestinal; NAC, N-Acetyl-L-Cysteine; MTT, methylthiazolyldiphenyl-tetrazolium bromide; GSP, glutathione; DPPH, 2,2-diphenyl-1-picrylhydrazyl; ABTS: 2,2′-azino-bis(3-ethylbenzothiazoline-6-sulfonic acid; HO·, hydroxide radical; MW, molecular weight; TCAH, protein hydrolysate of tuna cardiac arterial bulbs generated under the optimized enzymolysis conditions of pepsin; SC, supercoiled; O_2_^−^·, superoxide anion radical; OC, open circular; LIN, linear; NO, nitric oxide.
